# Death from the Effects of Cold Water Treatment

**Published:** 1846-10

**Authors:** 


*Death from the Effects of Cold Water Treatment.*—The London Lancet, for August, 1846, contains an account of a patient who was killed by the improper use of cold water, also the trial and conviction of the doctor for man-slaughter. It seems that the patient had congestion of the lungs, and applied to the Hydropathic Institution kept by one Dr. Ellis, who pronounced it a case of hepatitis of the sub-acute character, and put him in the sheets wet in cold water, which caused his death in a short time.

Upon making a post-mortem examination the lungs were found to be in a highly congested state, which was no doubt much increased by the application of the cold water treatment, causing the death of the patient; but the liver was not diseased, as the hydropathic man had said.

It is a great pity that some of the evils of this catch-penny system in this country, could not be brought to light. They tell of all their successful cases, but we never hear of their bad cases, by them. The idea of taking a delicate female with disease of the heart, or any other disease, it makes no difference with them, and placing her in cold wet sheets, and obliging her to drink four or five tumblers of cold water three times a day, is too absurd for sane people to believe to be the best mode of treatment; yet we find many following the plan, whom we believe to be sane on all other subjects. Cold bathing is an excellent thing for those in health, and should be used daily, but not in the manner that it is by the hydropaths. But as to drinking cold water, we should be very careful, for all kinds of drinks taken in excess are injurious to the system.—*New York Med. and Surg. Rep.*

